# Investigation of gelatin/multi-walled carbon nanotube nanocomposite films as packaging materials

**DOI:** 10.1002/fsn3.81

**Published:** 2013-12-20

**Authors:** Gholamreza Kavoosi, Seyed Mohammad Mahdi Dadfar, Seyed Mohammad Ali Dadfar, Farhad Ahmadi, Mehrdad Niakosari

**Affiliations:** 1 Institute of Biotechnology, Shiraz UniversityShiraz, 71441-65186, Iran; 2 Department of Food Science, Shiraz UniversityShiraz, 71441-65186, Iran; 3 Department of Chemical and Petroleum Engineering, Sharif University of TechnologyTehran, Iran; 4 Department of Animal Science, Shiraz UniversityShiraz, 71441-65186, Iran

**Keywords:** Antibacterial properties, gelatin, mechanical properties, MWCNT, water resistance

## Abstract

Gelatin composite films were prepared from gelatin solutions (10% w/v) containing multi-walled carbon nanotubes (MWCNT, 0.5, 1, 1.5, and 2% w/w gelatin) as nanofiller. The water solubility, water swelling, water uptake, water vapor permeability (WVP), mechanical, and antibacterial properties of the films were examined. Water solubility, water swelling, water uptake, and WVP for gelatin films were 45 ± 1%, 821 ± 42%, 45 ± 1.1%, and 0.4 ± 0.022 g mm/m^2^ kPa h, respectively. Incorporation of MWCNT caused a significant decrease in water solubility, water swelling, water uptake, and WVP. Gelatin/MWCNT films containing 1–1.5% MWCNT showed the lowest water vapor transmission. Tensile strength, elongation at break, and Young's modulus for gelatin films were 13.4 ± 1.2 MPa, 95 ± 5%, and 45.4 ± 7 MPa, respectively. Incorporation of MWCNT caused a significant increase in tensile strength and decrease in the elongation at break. The largest mechanical strength was found at 1.5% MWCNT. All gelatin/MWCNT films showed significant antibacterial activities against both gram-positive and gram-negative bacteria. Our results suggest that the gelatin/MWCNT composites films could be used as a very attractive alternative to traditional materials for different biomedical and food applications.

## Introduction

Gelatin (a denatured collagen peptide with MW = 100 kDa) is a soluble protein obtained by partial hydrolysis of collagen, the main insoluble fibrous protein constituent of bones, cartilages, and skins with high potential applications in food and pharmaceutical industries (Rawdkuen et al. 2010; Avena-Bustillos et al. 2011; Gomez-Guillen et al. 2011; Gan et al. 2012). The pharmaceutical applications of gelatin are based mainly on the gel/film-forming properties. Recently, an increasing number of new applications have been found for gelatin in products such as emulsifiers, foaming agents, colloid stabilizer, hydrogels, packaging materials, wound dressing, and micro-encapsulating agents (Gomez-Guillen et al. 2002; Boateng et al. 2008; Zhao et al. 2008; Arora and Padua 2010; Lucera et al. 2012). Due to the highly hydrophilic nature, gelatin shows relatively poor mechanical and barrier properties. Thus, the current trends in designing gelatin-based biodegradable materials for packaging and biomedical applications are focused on developing the films with improved mechanical and water resistance properties by combining gelatin with other biopolymers, synthetic polymer, nanofiller, plasticizer as well as cross-linker agents (Sorrentino et al. 2007; Cao et al. 2009; Gomes et al. 2013).

As suggested above, the use of gelatin-based biodegradable films has been strongly limited because of their poor mechanical properties. The incorporation of nanofillers into gelatin has been the topic of much research in order to overcome these advantages. Among these nanofillers carbon nanotubes are very attractive. Carbon nanotubes are some of the most attractive nanomaterials because of their unusual physicochemical, mechanical, and electrical properties. These materials are promising as novel nanomaterials for various biomedical applications because they can be used to deliver a variety of therapeutic agents and biomolecules to the target disease sites (Ivanova et al. 2012; Vardharajula et al. 2012). Although toxicity of carbon nanotubes appears still controversial in the literature, nano packaging is being designed to enable materials to interact with food, emitting antibacterial and antioxidants, and to alter the properties of food, including its texture, heat tolerance, and shelf life (Sorrentino et al. 2007; Tang et al. 2012). To solve the toxicity problem of nanofiller, nanostructured materials based on biodegradable and renewable polymers have become a topic of interest. For example, Sanchez-Garcia et al. (2010) reported that carbon nanotubes can be used to enhance the thermal and mechanical properties of polyhydroxybutyrate-co-valerate nanocomposite. Fama et al. (2011) and Wan et al. (2000) reported that multi-walled carbon nanotubes (MWCNT) increased ultimate tensile strength and tensile toughness of starch/MWCNT nanocomposites. Li et al. (2000, 2004) synthesized a novel gelatin-carbon nanotube hydrogel and reported that carbon nanotubes inhibited the swelling capacity of gel matrix. Similarly, Haider et al. (2007) suggested that incorporation of MWCNT gradually decreased the swelling of gelatin/MWCNT nanocomposite.

The aim of this work was to develop gelatin-based nanocomposite films reinforced by low concentrations of carbon nanotubes in order to obtain improvements in water solubility, water swelling, water uptake, water vapor permeability (WVP), tensile strength, elongation at break, Young's modulus, and antibacterial properties. The effects of the addition of MWCNT on the water solubility, water swelling, water uptake, WVP, mechanical, and antibacterial behavior of the nanocomposite were examined. Our results indicate that the nanocomposite obtained can be used in food and biomedical applications as an attractive alternative to traditional synthetic polymer.

## Material and Methods

### Materials

Bovine gelatin was purchased from Sigma-Aldrich (Saint Louis, MO, USA). MWCNTs were supplied by US Research Nanomaterials (Twig Leaf Lane, Houston, TX, USA). The diameter and length of the studied MWCNT were 10–20 nm and 20–50 *μ*m, respectively, and its purity was higher than 95%. MWCNT was dissolved in ethanol solution (50%) and sonicated using a sonication bath (Baldelin ultrasonoc bath, Berlin, Germany) at 40 W for 1 h at 50°C (Voge et al. 2013).

### Preparation of gelatin/MWCNT film-forming solutions and film casting

To prepare gelatin film-forming solutions, different concentrations of MWCNTs (0.5, 1, 1.5, and 2% w/w gelatin) were mixed with 10% (w/v) gelatin in deionized water and stirred for 12 h at 50°C. To avoid photochemical reactions all the solutions were kept in the dark. The gelatin/MWCN solutions were mixed and sonicated again using a sonication bath at 40 W for 1 h at 50°C and poured onto the polystyrene petri dish for film casting. The films were placed at room temperature until films were dried (Li et al. 2000, 2004). After drying, films were peeled off from the plate surface and left to equilibrate at relative humidity of 75% at 4°C in a closed box containing saturated salt solution of NaCl. The films were then ready for further tests. Thicknesses of films were measured to the nearest 0.01 mm using a micrometer (The L. S. Starrett Co. Ltd., Great Britain, U.K.) and the average was taken (in five spots of three films) 100 ± 5 *μ*m.

### Water solubility of gelatin/MWCNT films

The gelatin/MWCNT film cuts (20 mm × 20 mm ×0.10 mm) were placed in an oven (Fan Azma Gostar, Tehran, Iran) at 104°C for 24 h and then weighed. The weight at this condition was taken as initial weight (*W*_i_). Then, the dried films were immersed into 100-mL Erlenmeyer flask containing 50 mL of distilled water and placed inside the shaker for 24 h at 25°C (Jal Tajhiz Labtech. Co. Ltd., Tehran, Iran). Thereafter, the films were taken out and placed in an oven (Fan Azma Gostar) at 104°C for 24 h and then weighed. The weight at this condition was taken as final weight (*W*_f_). The percentage of weight loss was taken as water solubility (Wan et al. 2000; Haider et al. 2007). At least four tests were performed on two films and the average values were reported.

### Swelling test of gelatin/MWCNT films

The gelatin/MWCNT film cuts (20 mm × 20 mm ×0.10 mm) were dried in an oven (Fan Azma Gostar) at 104°C for 24 h and then weighed. The weight at this condition was taken as initial weight (*W*_i_). Each sample was immersed into a 100-mL Erlenmeyer flask containing 50 mL of the distilled water. The samples were kept at room temperature for 24 h. Each sample was taken out of the flask after 24 h, wiped between filter papers to remove the excess surface water and were weighed. The weight at this condition was used as final weight (*W*_f_). The percentage of weight gaining was taken as swelling percentage (Wan et al. 2000; Haider et al. 2007). At least four tests were performed on two films and the average values were reported.

### Water uptake of gelatin/MWCNT films

The gelatin/MWCNT film cuts (20 mm × 20 mm ×0.10 mm) were dried in an oven (Fan Azma Gostar) at 104°C for 24 h. The weight at this condition was taken as initial weight (*W*_i_). Then, films transferred into desiccators at 100% relative humidity at 37°C for about 1 week and allowed to absorb water until constant weight (equilibrium) was reached. Saturation condition was checked by observing no changes in successive weight uptake measurements by the film cuts. The weight at this condition (equilibrium state) was used as final weight (*W*_f_). The percentage of weight gaining was taken as water uptake (Wan et al. 2000; Haider et al. 2007). At least four tests were done on two films and the average values were reported.

### Water vapor permeability of gelatin/MWCNT films

The gelatin/MWCNT film cuts (7 cm diameter) were conditioned for 24 h at 25°C and 75% relative humidity. WVP of the film samples were examined using aluminum cups (height and diameter of 2.1 and 5.6 cm, respectively) filled with 20 g silica. The cups were covered with the film samples and placed at 25°C and 75% relative humidity in desiccators. The weight of the cups was measured at 3 h intervals during 1 day. A graph was plotted the mass change (g) against time (h). Water vapor transmission rates (WVTR) of the films were calculated from the slope of mass change (g) versus time (h) plots per film area (m^2^) and expressed as g/m^2^·h. The WVP was calculated using the following formula: WVP (g mm/m^2^ kPa h) = [(WVTR × *T*)]/Δ*P*. *T* is the film thickness (mm) and Δ*P* is the partial water vapor pressure difference (kPa) between the two sides of the film (4.2449 kPa at 30°C) (Ahmad et al. 2012; Nunez-Flores et al. 2012). Tests were done in hexaplicate on three films and average values and standard errors were provided.

### Mechanical properties of gelatin/MWCNT films

The gelatin/MWCNT film cuts (60 mm × 10 mm × 0.10 mm) containing different concentrations of MWCNT were placed in a closed container with relative humidity of 65% for 48 h. The tensile strength test was then performed by stretching the film at pretest, test, and posttest speeds of 1, 1, and 10 mm/min, respectively, using an Instron testing machine (Santam, Tehran, Iran). The nominal stress–strain curves were obtained and tensile strength (the maximum stress), Young's modulus (the initial slope of the stress-stain curves at the linear part), and elongation at break (where the film is torn) values were determined. The area of film used for each experiment was 60 mm × 10 mm. However, 20 mm of the films were within the jaws, so the initial length of the film was taken as 40 mm. The initial cross-sectional area of film cuts were 10 mm × 0.10 mm (Li et al. 2004; Fama et al. 2011). All the mechanical tests were carried out at room temperature. A minimum of four tests were performed on two films and the average values were reported.

### Light barrier property of gelatin/MWCNT films

The film cuts (1 cm × 6 cm) were directly placed against two sides of empty spectrophotometer covets and the UV–visible spectrophotometric measurements were carried out using Shimadzu UV–visible spectrophotometer (Kyoto, Japan) from 200 to 700 nm. The opacity of the films was calculated by the following equation: opacity (nm/mm) = *A*_440_/film thickness. *A*_440_ is the value of absorbance at 440 nm. A minimum of four tests were performed on two films and the average values were reported.

### Scanning electron microscopy

Scanning electron microscopy (SEM) of the gelatin and gelatin/MWCNT films was performed using a Hitachi 570 SEM (FESEM Hitachi S4160; Kyoto, Japan) in the School of Metallurgy and Materials Engineering University of Tehran, Tehran, Iran. The film samples (16 mm ×14 mm × 0.1 mm) were immersed in liquid nitrogen and cryo-fractured by hand. The samples fixed on the sample holder and then coated with gold. SEM pictures with 4000× magnification were taken with an accelerating voltage of 24 kV.

### Antibacterial assay of gelatin/MWCNT films using disk diffusion

All microorganisms were obtained from the Persian type culture collection (PTCC), Tehran, Iran. The films were individually tested against two gram-negative bacteria [*Pseudomonas aeruginosa* PTCC 1074 (ATCC 9027 and *Escherichia coli* PTCC 1330 (ATCC 8739)] and two gram-positive bacteria [*Staphylococcus aureus* PTCC 1112 (ATCC 6538) and *Bacillus subtilis* PTCC 1023 (ATCC 6633)]. To investigate the antimicrobial activity of the films using disk diffusion, 15 mm diameter disks (thickness of 0.10 mm) were cut from different parts of the films and sterilized by autoclaving for 30 min at 120°C (Ahmad et al. 2012; Nunez-Flores et al. 2012). Bacterial suspensions with a turbidity equivalent to a McFarland 0.5 standard were prepared at 10^8^ CFU/mL and then diluted to 10^5^ CFU/mL with Luria-Bertani (LB). The adjusted bacterial suspensions (0.1 mL) were spread onto the nutrient agar plates containing LB. Subsequently, the disks were placed in direct contact with the agar medium. Plates were incubated at 37°C for 24 h (incubator with ventilator; Pars Azma Co., Tehran, Iran). Films without MWCNT under the same condition were used as control. The diameters of clear inhibition zones, including the diameter of the disk, measured using a ruler and disks were used to evaluate antibacterial potential of films. At least six tests were performed on two films and the average was provided.

### Statistical analysis

Data are expressed as the means ± standard deviations of at least three independent experiments. The significant differences between treatments were analyzed by one-way analysis of variance (ANOVA) and Duncan's tests at *P* < 0.05 using statistical package for the social sciences (SPSS, Abaus Concepts, Berkeley, CA) software.

## Results and Discussion

### Solubility determination of films

The solubility percentage for gelatin films was 45% in the presence of aqueous liquid. Incorporation of 0.5–2% MWCNT caused a significant decrease (*P* < 0.05) in the solubility of gelatin films from 43% to 30.3%, dose-dependently (Table 1). Gelatin is a water-soluble material, which can partially dissolve when coming into contact with an aqueous medium especially for long period of time. The significant decrease in the water solubility of the gelatin films with loading of MWCNT is probably as a result of the hydrophobic nature of MWCNT (Yadav et al. 2010; Tonelli et al. 2012). The decrease in the solubility by incorporation of MWCNT is probably due to water resistance of carbon nanotubes material. This water resistance could be attributed to the highly crystalline and hydrophobic character of carbon nanotubes (Sanchez-Garcia et al. 2010). Hydrophobic domains of gelatin can essentially interact with the side walls of the nanotubes through hydrophobic interaction and thereby cross-linking density between the gelatin and MWCNT was increased. These interactions strengthened the gelatin matrix and subsequently caused a decrease in its solubility (Li et al. 2000, 2004).

**Table 1:** Water solubility, swelling, water uptake, and water vapor permeability of gelatin films incorporated with multi-wall carbon nanotube (MWCNT).

Films	Solubility (%)	Swelling (%)	Water uptake (%)	WVP (g mm/kPa m^2^ h)
Gelatin	45 ± 1.0^a^	821 ± 42^a^	45 ± 1.1^a^	0.4 ± 0.022^a^
Gelatin + MWCNT 0.5%	43 ± 1.2^ab^	751 ± 25^ab^	39 ± 1.2^b^	0.33 ± 0.021^b^
Gelatin + MWCNT 1%	39 ± 1.5^b^	713 ± 19^bc^	37.4 ± 0.8^bc^	0.28 ± 0.014^c^
Gelatin + MWCNT 1.5%	35 ± 1^c^	677 ± 12^cd^	36 ± 0.9^cd^	0.30 ± 0.013^c^
Gelatin + MWCNT 2%	30.3 ± 0.9^d^	645 ± 25^d^	33 ± 1.2^c^	0.340 ± 0.011^b^

Mean values with different letters within a column are significantly different as analyzed by Duncan's multiple range tests at (*P *<* *0.05).

### Swelling capacity and water uptake of films

The swelling and water uptake for gelatin films were 821% and 45%, respectively. Incorporation of 0.5% to 2% MWCNT caused a significant decrease (*P* < 0.05) in the swelling (from 751% to 645%) and water uptake (from 39% to 33%) of the gelatin films (Table 1). Gelatin is a hydrophilic material that is expected to absorb molecules of water. Incorporation of MWCNT reduced swelling and water uptake capacity of the gelatin films possibly due to increase in hydrophobicity (water resistance) of the gelatin matrix. This phenomenon might be due to the hydrophobic effect of the MWCNT and an increase in cross-linking density between the MWCNTs and gelatin (Li et al. 2000, 2004). Hydrophobic domains of gelatin can essentially interact with the nanotubes through hydrophobic interaction. This event saturates gelatin network with MWCNT, consequently water molecules cannot diffuse to the gelatin network, thereby swelling and water uptake decreased (Haider et al. 2007; Sanchez-Garcia et al. 2010).

### Water vapor permeability of films

The WVP for gelatin films was 0.4 g mm/kPa m^2^ h. Incorporation of 0.5% to 1% MWCNT caused a significant decrease (*P* < 0.05) in WVP of the gelatin films from 0.33 to 0.28 g mm/kPa m^2^ h. Incorporation of 1% to 2% MWCNT caused a significant increase (*P* < 0.05) in WVP of the gelatin films from 0.28 to 0.34 g mm/kPa m^2^ h. At 1–1.5% of MWCNT the lowest WVP was obtained (Table 1). Gelatin is a hydrophilic material, so it strongly interacts with water molecules and causes a reduction in the water vapor transmission through gelatin matrix. Our results suggested that MWCNTs had bi-functional effects on the water vapor transmission across gelatin matrix. Addition of MWCNT at low concentrations (0.5–1% of gelatin powder) could introduce a twisted path for water molecules to pass through and increase WVP (Li et al. 2000; Sanchez-Garcia et al. 2010). At moderate concentrations (1.5%), MWCNT probably dispersed well in the gelatin matrix (as confirmed by SEM analysis) and blocked the water vapor transmission. At higher concentrations (2%), carbon nanotubes might agglomerate which in turn could decrease the effective contents of the MWCNT and facilitates the water vapor penetration (Li et al. 2004; Haider et al. 2007).

### Mechanical properties of the films

Tensile strength, elongation at break, and Young's modulus for gelatin films were 13.4 MPa, 95%, and 45.4 MPa, respectively (Table 2). Incorporation of 1–1.5% MWCNT caused a significant increase (*P* < 0.05) in tensile strength (from 22.6 to 36.5 MPA), decrease (*P* < 0.05) in elongation at break (from 66% to 44.5%), and increase (*P* < 0.05) in Young's modulus (from 71.5 to 110 MPa). Incorporation of 1.5–2% MWCNT caused no significant decrease (*P* > 0.05) in tensile strength (from 36.5 to 30.3 MPa), a significant increase (*P* < 0.05) in elongation at break (from 44.5% to 55.2%), and a no significant decrease (*P* > 0.05) in Young's modulus (from 110 to 93 MPa). At 1.5% of MWCNT the largest tensile strength and Young's modulus was obtained (Table 2). The increment in the mechanical properties of the gelatin/MWCT (at 1.5%) is probably due to the substantial dispersion of MWCNTs in the gelatin matrix and cross-linking efficiency between the matrix (gelatin) and filler (MWCNT). Thus, efficient load transfer from the gelatin chain to the carbon nanotubes caused the idea that the high mechanical strength of the nanotubes was transferred to the gelatin matrix (Pan et al. 2012; Voge et al. 2013). Loading high concentrations of MWCNT (2%) to gelatin/MWCNT films caused a significant decrease in the mechanical properties of the films probably, due to the agglomeration of MWCNT. These agglomerates possibly lowered interaction between gelatin chains, and may hinder gelatin chain-to-chain interactions and consequently, cause a decrease in tensile strength of the film (Nabeta and Sano 2005; Momota et al. 2010; Sanchez-Garcia et al. 2010). Poly (butylenes succinate)/MWCNT nanocomposite consisting of carbon nanotubes also exhibited enhanced mechanical properties (Shih et al. 2008). Small quantities of carbon nanotubes can improve the mechanical properties of poly(ethylene-2, 6-naphthalate)/carbon nanotubes composite (Kim et al. 2009). Furthermore, incorporation of small quantities of MWCNT into the starch/MWCNT nanocomposite caused highly improved tensile properties (Fama et al. 2011), which are in accordance with our experimental results.

**Table 2:** Tensile strength, elongation at break, Young's modulus, and opacity of gelatin films incorporated with multi-wall carbon nanotubes (MWCNT).

Films	Tensile strength (MPa)	Elongation at break (%)	Young's modulus (MPa)	Opacity (nm/mm)
Gelatin	13.4 ± 1.2^d^	95 ± 5^a^	45.4 ± 7^c^	5.2 ± 0.95^d^
Gelatin + MWCNT 0.5%	22.6 ± 3.2^c^	66 ± 4.7^b^	71.5 ± 6.4^b^	7 ± 0.54^cd^
Gelatin + MWCNT 1%	29.2 ± 2.1^bc^	49 ± 6^c^	86.5 ± 11.5^ab^	8.4 ± 0.78^bc^
Gelatin + MWCNT 1.5%	36.5 ± 2.7^a^	44.5 ± 3.4^c^	110 ± 8.5^a^	9.5 ± 1.1^ab^
Gelatin + MWCNT 2%	30.3 ± 3.0^bc^	55.2 ± 2.6^c^	93 ± 9.ab^a^	11.4 ± 1.4^a^

Mean values with different letters within a column are significantly different by Duncan's multiple range tests at (*P *<* *0.05).

### Light absorbance of gelatin films

**Figure 1:**
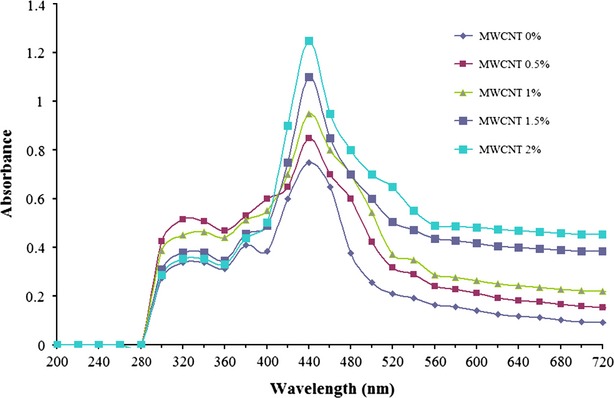
Light absorbance of gelatin/MWCNT composite films.

Light absorbance images of the films at wavelengths between 200 and 700 nm are shown in Figure 1. Gelatin films showed light absorbance in the range between 280 and 480 nm, while maximum absorbance was at 440 nm. The opacity (cloudiness) of gelatin films is shown in Table 2. Incorporation of MWCNTs in the gelatin films caused a significant increase in the light absorbance and opacity of the films. Protein-based films are considered to have high light absorbance properties, owing to their high content of aromatic amino acids which absorb UV light (Ahmad et al. 2012). The addition of MWCNTs increased greatly the opacity of gelatin films. Thus, the gelatin films lost their typical transparent and colorless appearance. However, the resulting gelatin/MWCNT composite films gained light barrier properties, which could be interesting in certain food and biomedical applications for preventing UV-induced lipid peroxidation (Voge et al. 2013). The increase in the light absorbance more likely depended on the distribution of MWCNT in the gelatin matrix as well as the interaction between MWCNT and gelatin. This effect led to differences in film matrix morphology with different light absorbance. Furthermore, MWCNT particles that were localized in the gelatin matrix increased the opacity of the gelatin film, more likely due to the light scattering effect (Shih et al. 2008).

### Film morphology

**Figure 2:**
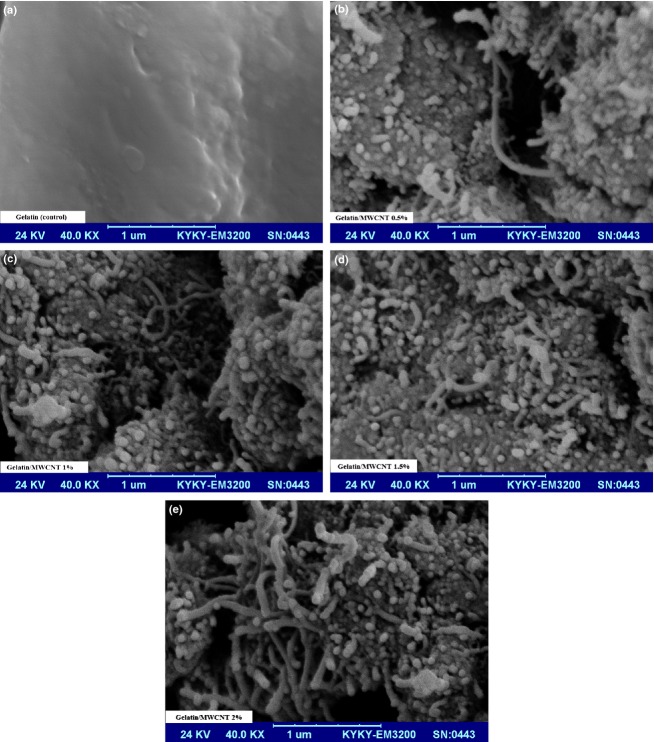
Scanning electron microscopy images of gelatin/MWCNT composite films.

Scanning electron micrographs of gelatin/MWCNTs composite films are presented in Figure 2. MWCNT (at 1.5%) showed good dispersion in the gelatin matrix with no evidence of agglomeration. Due to hydrophobic nature of carbon nanotubes, the homogenous dispersion of MWCNT in the gelatin is difficult. In order to obtain a homogeneous dispersion, MWCNT can be dispersed in the gelatin solution through sonication. This homogenous dispersion can increase tensile strength while it can reduce water vapor transmission (Haider et al. 2007; Fama et al. 2011).

### Antibacterial assay of films

The results of disk diffusion are summarized in Table 3. The initial diameter of all films was fixed at 15 mm. The diameters of clear inhibition zones were used for antibacterial activity analysis. According to the results obtained, all the gelatin films without MWCNT showed no activity against the tested bacteria. For the gelatin films containing different MWCNT concentrations inhibitory zones were clear. The results of disk diffusion indicated that gelatin/MWCNT is effective to both gram-positive bacteria and gram-negative bacteria. Ali-Boucetta and other and Kang and coworker showed that carbon nanotubes exhibit significant antibacterial activity to *E. coli* through cell membrane damage and expression of stress-related gene products (Kang et al. 2008; Ali-Boucetta et al. 2011). Other studies also reported that, carbon nanotubes exhibited significant antibacterial activity to *B. anthracis* through cell membrane damage and cell inactivation (Pangule et al. 2010; Aferchich et al. 2012; Lilly et al. 2012). Aslan and others and Meredith and colleague suggested that carbon nanotubes are relatively flexible and interact with cell membranes and penetrate various microorganisms and consequently cause cell death (Aslan et al. 2012; Meredith et al. 2013).

**Table 3:** The antibacterial activity of gelatin films incorporated with multi-wall carbon nanotubes (MWCNT).

	Inhibition zone diameter (mm)			
Films	*Bacillus subtilis*	*Staphylococcus aureus*	*Escherichia coli*	*Pseudomonas aeruginosa*
Gelatin	0 ± 0^d^	0 ± 0^d^	0 ± 0^d^	0 ± 0^d^
Gelatin + MWCNT 0.5%	15 ± 0.2^c^	15 ± 0.1^c^	15 ± 0.2^c^	15 ± 0.1^c^
Gelatin + MWCNT 1%	16.5 ± 0.3^b^	16.2 ± 0.4^b^	15.6 ± 0.2^b^	15.3 ± 0.4^bc^
Gelatin + MWCNT 1.5%	17.4 ± 0.7^ab^	17.5 ± 0.6^ab^	16.4 ± 0.6^ab^	15.8 ± 0.5^b^
Gelatin + MWCNT 2%	18.2 ± 0.8^a^	18.3 ± 0.8^a^	17.2 ± 0.7^a^	16.8 ± 0.3^a^

Antibacterial activity was expressed as diameter of bacterial growth inhibition zone in the presence of gelatin films incorporated with MWCNT. Mean values with different letters within a column are significantly different as analyzed by Duncan's multiple range tests at (*P *<* *0.05).

## Conclusion

Gelatin/MWCNT composite films were prepared by dissolving carbon nanotubes in aqueous gelatin solutions. The water solubility, water swelling, water uptake, WVP tensile strength, elongation at break, and Young's modulus properties and the film morphology were studied. The composite films showed decrease in water solubility, water swelling, water uptake, WVP as compared to gelatin films. This showed that carbon nanotubes inhibited the solubility and swelling capacity probably by hydrophobic interaction between MWCN and gelatin. The lowest water transmission was found at 1–1.5% of MWCNT. Incorporation of MWCNT also caused a significant increase in tensile strength, decrease in elongation at break, and an increase in the Young's modulus of the films. The largest significant increase in mechanical strength was found at 1.5% of MWCNT that might be due to well-dispersed nanotubes in the gelatin matrix and strong adhesion between them. MWCNT particles that were localized in the gelatin matrix increased the opacity of the gelatin film, more likely due to the light scattering effect of carbon nanotubes. SEM observations indicate that carbon nanotubes (at 1.5%) were well dispersed in the gelatin matrix and good adhesion between them was obtained that led to an increase in the tensile strength and a decrease in water vapor transmission. All gelatin/MWCNT films showed significant antibacterial activities in a dose-dependent manner against both gram-positive and gram-negative bacteria. In summary, the improved water binding capacity, water permeation, mechanical, and light barrier properties as well as antibacterial activity suggest a great potential of biodegradable and biocompatible gelatin/MWCNT nanocomposite films, in applications including biomedicine, food, and beverage packaging.
